# Increased Risk of Acute Coronary Syndrome among Patients with Urinary Stone Disease: A Nationwide Population-Based Cohort Study

**DOI:** 10.1371/journal.pone.0102349

**Published:** 2014-07-10

**Authors:** Shun-Fa Hung, Chao-Yuan Huang, Cheng-Li Lin, Shiu-Dong Chung, Chi-Jung Chung, Chia-Hung Kao, Chao-Hsiang Chang

**Affiliations:** 1 Division of Urology, Department of Surgery, Far Eastern Memorial Hospital, Ban Ciao, Taipei, Taiwan; 2 Graduate Institute of Clinical Medicine, College of Medicine, Fu-Jen Catholic University, New Taipei, Taiwan; 3 Department of Urology, National Taiwan University Hospital, Taipei, Taiwan; 4 Management Office for Health Data, China Medical University Hospital, Taichung, Taiwan; 5 College of Medicine, China Medical University, Taichung, Taiwan; 6 Division of Urology, Department of Surgery, Far Eastern Memorial Hospital, New Taipei, Taiwan; 7 School of Medicine, College of Medicine, Fu Jen Catholic University, Taipei, Taiwan; 8 Department of Urology, Buddhist Tzu Chi General Hospital and Tzu Chi University, Hualien, Taiwan; 9 Department of Health Risk Management, College of Public Health, China Medical University, Taichung, Taiwan; 10 Department of Medical Research, China Medical University Hospital, Taichung, Taiwan; 11 Graduate Institute of Clinical Medical Science and School of Medicine, College of Medicine, China Medical University, Taichung, Taiwan; 12 Department of Nuclear Medicine and PET Center, China Medical University Hospital, Taichung, Taiwan; 13 Department of Urology, China Medical University and Hospital, Taichung, Taiwan; University of Bologna, Italy

## Abstract

**Background/Objectives:**

Urinary stones (US) are associated with systemic metabolic and endocrine disorders that share risk factors typically associated with acute coronary syndrome (ACS).

**Methods:**

For this investigation, 30 142 patients with US were set as the research group, and 121 768 randomly selected patients were set as the comparison group through frequency matching by age, sex, and index year. Each patient was individually tracked to identify those who developed ACS during the follow-up period. Cox proportional hazards regression and the Kaplan-Meier method were adopted to calculate the hazard ratios of ACS risk and plot the survival curve.

**Results:**

Overall, 275 (13.4 per 10 000 person-y) and 736 events (9.1 per 10 000 person-y) were observed among patients in the research and comparison cohorts, respectively. The patients with US had a substantially lower ACS-free survival rate compared with that of the patients in the comparison cohort (*P*<.001). After adjusting for potential risk factors, the patients with US were observed to have a 1.22-fold higher risk of ACS compared with patients in the comparison cohort (95% confidence interval = 1.05–1.40, *P*<.001), particularly among younger patients.

**Conclusions:**

The results indicate that US is associated with increased risk of developing ACS, particularly among young (≤49 years) and male adults. Future studies should examine the possible mechanisms of US-related ACS morbidity by conducting multicenter recruitment and measurements of laboratory data.

## Introduction

Acute coronary syndrome (ACS) includes ST-segment elevation myocardial infarction (STEMI), non-STEMI (NSTEMI), or unstable angina [1]. The pathomechanism is typically associated with rupture of an atherosclerotic plaque, and partial or complete thrombosis of the infarct-related artery [2]. Previous research has estimated that more than 1 million hospital admissions in the United States are due to ACS [3]. Because of the high mortality and reinfarction rates, ACS is considered a major public health concern [4]. Relevant evidence indicates that the prevalence of ACS increases with age, and is associated with risk factors such as hypertension, diabetes mellitus, hyperlipidemia, and smoking [5]–[8].

Urinary stone (US) disease is a relatively prevalent disease in western countries [9]. Increasing epidemiological evidence supports an association between urinary tract calculus and obesity, diabetes mellitus, and hypertension [10]–[15]. These associated chronic medical disorders are major characteristics of the metabolic syndrome (MS), which results specifically from insulin resistance and increases the risk of ACS [16], [17]. The high prevalence of US-diagnosed patients with MS indicates that insulin resistance might be involved in the pathophysiology of stone disease [18], [19]. Collectively, both US and ACS are recognized as systemic diseases and associated with insulin resistance and metabolic disorders. However, research on the relationship between ACS and US is scarce. Therefore, using a nationwide population-based dataset, we examined the influence of US on the incidence of ACS.

## Materials and Methods

### Data source

Taiwan launched a single-payer National Health Insurance program in March 1995 that was set up by the Bureau of National Health Insurance (http://www.nhi.gov.tw/english/index.aspx). Since 2000, the National Health Insurance Research Databases (NHIRDs) have been established and managed by the National Health Research Institutes (NHRI) in Taiwan. The NHIRDs, a medical claims database (included inpatient and outpatient claim records, medical treatment and etc.) and personal information (included gender, birthday, residential area and etc.), has been released for research purposes. Longitudinal Health Insurance Database 2000 (LHID2000) is composed of historical claims data for one million patients who are randomly selected from NHIRDs. The NHRI encrypts personal identification numbers to ensure privacy protection and provides researchers with anonymous identification numbers by linking claims data. Disease was defined according to the International Classification of Diseases, 9th Revision, Clinical Modification (ICD-9-CM) in the database. The NHRID encrypts the patients’ personal information for privacy protection and provides researchers with anonymous identification numbers associated with the relevant claim information, which includes the patient’s sex, date of birth, registry of medical services, and medication prescriptions. Patient consent is not required for accessing the NHIRD.

### Data Availability Statement

All data and related metadata are deposited in an appropriate public repository: The study population’s data were from Taiwan NHIRD (http://w3.nhri.org.tw/nhird//date_01.html) are maintained by Taiwan National Health Research Institutes (http://nhird.nhri.org.tw/) [20]. The National Health Research Institutes (NHRI) is a non-profit foundation established by the government.

### Ethics statement

Because identification numbers of patients had been encrypted, patient consent was not required for this study. This study was approved by the Research Ethic Committee at China Medical University (CMU-REC-101-012). The committee waived the requirement for consent.

### Study participants

The section criteria for inclusion in this study are as follows: (1) patients aged more than or equal to 20 years; and (2) newly diagnosed urinary stones (US) (ICD-9-CM codes 592.0, 592.1, 594.0 and 594.1). Patients with previous acute coronary syndrome (ACS) (ICD-9-CM code 410 and 411.1) or incomplete information were excluded. We identified 30,442 patients as a US cohort and used the date of diagnosed US as the index date. Four controls were randomly selected from insured people without US and ACS history before the index date, and they were excluded with the same criteria of the US cohort. The non-US cohort was matched with US cohort based on age category (every 5 years each), sex, and the year of index date. The non-US cohort included 121,768 patients.

### Outcome definition

Both cohorts were followed until ACS was diagnosed or censored because of death, loss to follow-up, termination of insurance, or the end of 2010. The comorbidities that might associate with ACS to be included in this study were hypertension (ICD-9-CM codes 401–405), diabetes (ICD-9-CM codes 250), hyperlipidemia (ICD-9-CM codes 272), cerebrovascular disease (CVA) (ICD-9-CM codes 430–438), chronic obstructive pulmonary disease (COPD) (ICD-9-CM codes 490–496), end-stage renal disease (ESRD) (ICD-9-CM code 585), and urinary tract infection (UTI) (ICD-9-CM codes 590 and 595), identified at the baseline.

### Statistical analysis

The Chi-square test and t test were used to test the differences in categorical variables included age (≤49, 50–64 and >64 years), sex, income less than New Taiwan Dollar [(NTD) 15,000, 15,000–19,999, and more than 20,000/per month], occupation (white collar, blue collar and other), comorbidities and continuous variables contained age between two cohorts, respectively. The sex-, age-, income- and occupation-specific incidence densities of ACS were estimated in both cohorts. Crude and adjusted hazards ratios (HRs) and 95% confidence intervals (CIs) for ACS in the US cohort compared with the non-US cohort were estimated using univariable and multivariable Cox proportional hazard regression models. The multivariable models were simultaneously adjusted for demographic characteristics, monthly income (NTD), occupation and co-morbidities. Further, the Cox model was also used to estimate the HR of ACS associated with the cumulative frequency of medical visits by US, compared to the non-US cohort. The ACS-free survival rates were estimated using the Kaplan-Meier method, and the difference between the US cohort and non-US cohort was compared using log-rank test. Data were analyzed using SAS statistical software for Windows (version 9.2; SAS Institute Inc, Cary, NC), and the significance level was set at 0.05.

## Results

In this study, 30,442 diagnosed US patients at baseline were included and matched to 121,768 controls. Of the 152,210 sampled patients, the mean age was 48.3 and 48.8 for US patients and controls, respectively. Demographic characteristic data are shown in Table 1. The US cohort included more men than women (68.7%) and, because the cohorts were matched on sex, so too did the control cohort. Approximately 56% of participants were ≤49 years of age. Patients with US had a higher prevalence of all baseline comorbidities (P<0.0001) except ESRD, which is higher in non-US group (P<0.001). The US patients were more likely to have comorbidities of hyperlipidemia (20.3% vs. 13.3%, *p*<0.001), diabetes (12.9% vs. 9.6%, *p*<0.001), hypertension (43.0% vs. 30.8%, *p*<0.001), CVA (7.7% vs. 6.4%, *p*<0.001), COPD (26.0% vs. 19.8%, *p*<0.001), UTI (18.7% vs. 7.5%, *p*<0.001) and less to have ESRD (0.11% vs. 0.32%, *p*<0.001) than controls. In addition, non-US patients had a greater tendency to have monthly income ≤ NT$15,000 (*p*<0.001) and be employed as white collar occupation (*p*<0.001) compared to US patients.

**Table 1:** Comparison of demographics and comorbidity between urinary stones patients and non- urinary stones controls.

	Urinary stones				
	No (N = 121768)		Yes (N = 30442)		
	n	%	n	%	p-value
Age, year					
≤49	68236	56.0	17059	56.0	0.99
50–64	35188	28.9	8797	28.9	
>64	18344	15.1	4586	15.1	
Mean (SD)†	48.3	14.8	48.8	14.3	
Sex					
Female	38100	31.3	9525	31.3	0.99
Male	83668	68.7	20917	68.7	
Monthly Income (NTD)					
<15,000	26202	21.5	5746	18.9	<0.0001
15,000–19,999	55175	45.3	14406	47.3	
≥20,000	40391	33.2	10290	33.8	
Occupation					
White collar	64827	53.2	15569	51.1	<0.0001
Blue collar	40406	33.2	11109	36.5	
Others#	16535	13.6	3764	12.4	
Comorbidity					
Diabetes	11632	9.55	3929	12.9	<0.0001
Hypertension	26346	21.6	9277	30.5	<0.0001
Hyperlipidemia	16148	13.3	6166	20.3	<0.0001
CVA	7730	6.35	2341	7.69	<0.0001
COPD	24057	19.8	7927	26.0	<0.0001
UTI	9119	7.49	5684	18.7	<0.0001
ESRD	387	0.32	34	0.11	<0.0001

Chi-square test;

† T-test.

# Other occupations included primarily retired, unemployed, or low income populations.

**Figure 1:**
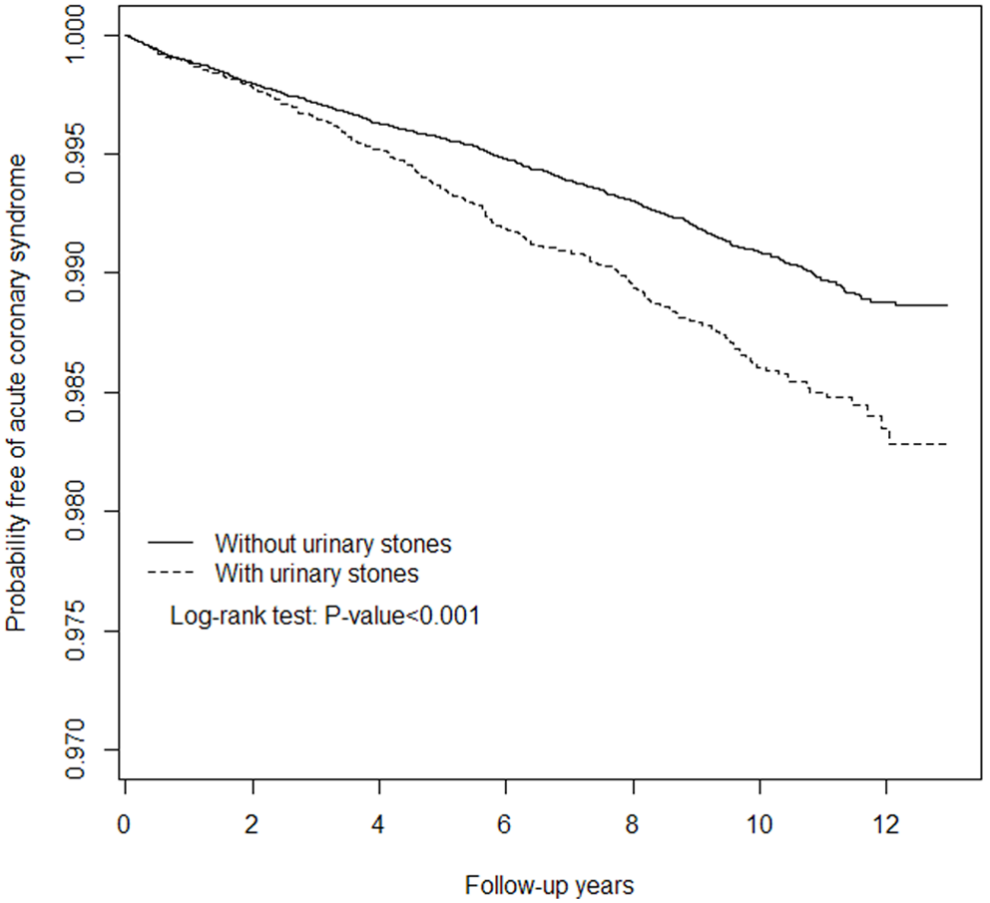
Probability free of acute coronary syndrome for patients with (dashed line) or without (solid line) urinary stones.

Table 2 shows the incidence rate of ACS and adjusted hazard ratio (HR) among the US cohort and the comparison cohort. The overall incidence rate of ACS was 1.47-fold higher in the US cohort than in the comparison cohort (13.4 vs. 9.12 per 1000 person-years). Kaplan–Meier survival analysis showed that patients with US had significantly higher rates of developing ACS than their comparative cohorts (log-rank test *p*<0.001; Figure 1). After adjusting for patients’ monthly income, occupation, hypertension, diabetes, CVA, ESRD, COPD, UTI and hyperlipidemia, the HR in US patients are at higher risk to develop subsequent ACS was 1.22 (95% CI 1.05–1.40) (Table 2). The sex-specific analysis shows the incidence of ACS was greater in men in both cohorts and men in the US cohorts had the greater HR than men in the non-US cohort (adjusted HR = 1.25, 95% CI = 1.06–1.47). Table 2 also shows the further analyses of the association of US with ACS according to age group. It shows that the HRs of ACS between US patients and controls were higher in younger groups. In the age group <49, the adjusted HR was as high as 1.76 (95% CI, 1.33–2.33; *p*<0.001), for those with US compared to controls. However, among the age groups 50–64 and >64, there was no statistical difference in the proportion of ACS between US patients and controls. In addition, US patients with white collar occupation had the highest adjusted HR of 1.28 (95% CI = 1.02–1.59).

**Table 2:** Incidence and adjusted hazard ratio of ACS stratified by sex, and age compared between with urinary stones and without urinary stones.

	Urinary stones						Compared to non-US	
	No			Yes				
Variables	Event	PY	Rate^#^	Event	PY	Rate^#^	Crude HR^*^(95% CI)	Adjusted HR^†^(95% CI)
All	736	807017	9.12	275	205894	13.4	1.47(1.28, 1.68)***	1.22(1.05, 1.40)**
Sex								
Female	185	255790	7.23	67	64526	10.4	1.44(1.09, 1.90)*	1.13(0.85, 1.51)
Male	551	551228	10.0	208	141368	14.7	1.47(1.25, 1.73)***	1.25(1.06, 1.47)**
Age								
≤49	139	478225	2.91	82	122156	6.71	2.30(1.75, 3.02)***	1.76(1.33, 2.33)***
50–64	307	226582	13.6	116	57183	20.3	1.50(1.21, 1.85)***	1.17(0.94, 1.46)
>64	290	102210	28.4	77	26556	29.0	1.03(0.80, 1.32)	0.91(0.71, 1.18)
Monthly Income (NTD)								
<15,000	177	163514	10.8	53	36969	14.3	1.32(0.97, 1.80)	1.12(0.82, 1.53)
15,000–19,999	354	365604	9.68	141	97767	14.4	1.49(1.23, 1.81)***	1.20(0.99, 1.47)
≥20,000	205	277899	7.38	81	71158	11.4	1.54(1.19, 2.00)***	1.29(1.00, 1.68)
Occupation								
White collar	308	427597	7.20	114	104247	10.9	1.52(1.23, 1.88)***	1.28(1.02, 1.59)*
Blue collar	307	272019	11.3	129	76366	16.9	1.50(1.22, 1.84)***	1.24(1.00, 1.53)*
Others^#^	121	107402	11.3	32	25281	12.7	1.13(0.76, 1.66)	0.94(0.63, 1.40)

Rate^#^, incidence rate, per 10,000 person-years; Crude HR^*^, related hazard ratio; Adjusted HR^†^: multivariable analysis including age, sex, monthly income (NTD), occupation and co-morbidities; *p<0.05, **p<0.01, ***p<0.0001.

Table 3 shows the further analyses of the association of US with ACS according to medical visit frequency for US. It shows that the HRs of ACS between study patients and controls were higher in those who visited for US more than 3 times per year. The adjusted HR was as high as 3.91 (95% CI, 3.14–4.87; *p*<0.001), for those with high visit frequency for US compared to controls.

**Table 3:** The risk of ACS among frequency for medical visits of stones in Cox proportional hazard regression.

Frequency for medical visit, per 1 years	Event	PY	Rate^#^	Crude HR^*^(95% CI)	Adjusted HR^†^ (95% CI)
None- urinary stones	736	807017	9.12	1.00	1.00
≤3	183	189711	9.65	1.06(0.90, 1.24)	0.92(0.78, 1.08)
>3	92	16183	56.9	6.23(5.01, 7.75)***	3.91(3.14, 4.87)***
p for trend					<0.0001

Rate^#^, incidence rate, per 10,000 person-years; Crude HR^*^, related hazard ratio; Adjusted HR^†^: multivariable analysis including age, sex, and co-morbidities; *p<0.05, **p<0.01, ***p<0.0001.

## Discussion

In this investigation, data of 30 442 US-diagnosed patients and 121 768 patients selected as the comparison cohort were analyzed. After adjusting for age, sex, and other potential risk factors, we observed that the risk of developing ACS during a 10-year follow-up period was 1.22-fold higher for the US-diagnosed cohort. Furthermore, we analyzed the association for various age groups, and identified significant associations between US and ACS, particularly among younger adults. To our knowledge, this is the first investigation to report an association between US and ACS based on a nationwide population-based survey, and the first to suggest that a higher frequency of medical visits related to US is associated with a higher risk of ACS.

US disease is common in the United States, affecting approximately 10.6% of men and 7.1% of women, and its prevalence continues to increase [9]. Numerous studies have indicated an association between stone disease various cardiovascular (CV) risk factors, including hypertension, diabetes, and metabolic syndrome [10]–[15], [21]. According to the CARDIA study, Reiner et al reported a relatively higher prevalence of subclinical atherosclerosis among young adults with a history of kidney stone disease [22], which is supported by the findings of this research. Specifically, we observed that young adults with US are at increased risk of developing subsequent ACS.

According to the National Cholesterol Education Program Adult Treatment Panel III, the metabolic syndrome (MS) comprises criteria related to obesity, hyperglycemia, dyslipidemia, and hypertension (HTN) [17]. Previous research has estimated that the prevalence of MS in the United States is approximately 25%, and increasing [16]. In addition to diabetes and cardiovascular risks, MS has been associated with US disease in recent studies [21], [23]. Kohjimoto et al reported that the probability of recurring or multiple stones among patients with 4 MS traits was up to 1.8-fold greater than that for patients without MS traits [24]. Their findings indicated that the clustering of MS traits is associated with increased risk of kidney stone formation. Epidemiological and basic studies have also provided strong indications regarding the association between MS and ACS. The primary features of MS, including hyperglycemia and insulin resistance resulting in systemic chronic inflammation, can lead to endothelial damage and atherosclerosis. In this investigation, the incidence of diabetes in the US-diagnosed cohort was considerably higher than that in the control group. A recent study reported an association between diabetic severity measured according to HbA1c and nephrolithiasis [25]. Weinberg et al estimated that over one million episodes of kidney stones in the United States might be attributable to type 2 diabetes, particularly among patients with poor glucose control [25]. Hyperglycemia and glucosuria might alter renal handling of calcium, phosphorus, and uric acid. Among patients with diabetes, hypercalciuria can be noted. In addition, insulin resistance is associated with abnormality in renal ammonium production, increased urinary acidification, hypocitraturia, and hypercalciuria. Previous studies have suggested insulin resistance as a potential cause of tubular calcium resorption, which leads to hypercalciuria [26]–[28]. Insulin resistance has also been associated with endogenous oxalate synthesis, which is a mechanism of stone formation [26]–[28].

These metabolic findings are associated with the development of uric acid and calcium stones. Consequently, both hyperoxaluria and hypercalciuria resulting from poor glycemic control could lead to the formation of calcium oxalate stones. Additionally, uric acid has been reported to be the main component of stones in a considerably high proportion of patients with diabetes [18]. Several studies have shown that uric acid excretion increased in the presence of hyperglycemia and glycosuria, a mechanism that might explain the increased formation of uric acid stones among diabetic patients [29], [30]. Rendina et al reported that MS patients are twice as likely to develop renal stones [31]. The Third National Health and Nutrition Examination Survey analysis showed that the probability of self-reported US disease is approximately twice as high among patients with MS than those without [21]. Consistent with the findings of Kohjimoto et al [24], a high number of MS components are associated with an increased risk of developing stone disease [21]. A plausible explanation for the association between MS and nephrolithiasis is that MS is associated with lower urine pH, which is an established characteristic of uric acid urolithiasis and a high risk factor for nephrolithiasis [32]–[34].

Several epidemiological studies have identified a link between urolithiasis and coronary heart disease. Domingos et al evaluated 23 346 patients and reported a statistically significant association between a self-reported history of kidney stones and myocardial infarction (odds ratio [OR] = 1.34; 95% CI = 1.00–1.79) and stroke (OR = 1.33; 95% CI = 1.02–1.74) after adjusting for age and body mass index (BMI) [35]. However, following multivariable adjustment, only myocardial infarction was significantly associated with US in women. Another study, using the cohort of Olmstead County in Minnesota, enrolled 4564 patients with kidney stones or ureter stones at the baseline who had not previously sustained a myocardial infarction [36]. Compared with 14 144 matched nonstone formers after a mean follow-up period of 9 years, the HR for experiencing a myocardial infarction was 1.31 for stone formers after adjusting for age and sex. The ratio increased to 1.35 after adjusting for other comorbidities related to coronary heart disease, including hypertension, diabetes, obesity, chronic kidney disease, gout, dyslipidemia, and tobacco use [36]. A case-control study conducted by Aydin et al indicated that patients with calcium oxalate nephrolithiasis exhibited a higher Framingham and Systemic Coronary Risk Evaluation (SCORE) scores compared with the age- and sex-matched controls [37]. They suggested that patients with calcium oxalate urolithiasis were at a higher risk of cardiovascular disease and mortality. However, the significant relationship is inconsistent with the findings of other studies. Tang et al examined the relationship between kidney stone disease, all-cause mortality, and CV mortality among US-diagnosed adults by analyzing data from the Third National Health and Nutrition Examination Survey for 1988–1994. However, no independent association of prevalent kidney stone disease with all-cause and CV mortality was identified [38]. A recent large-scale prospective study conducted in the United States employed data from patients (45 748 men, 196 357 women) with no history of CHD at the baseline. These patients had participated in the Health Professionals Follow-up Study, Nurses’ Health Study I (NHS I) (90 235 women, age = 30–55 y; follow-up period from 1992 to 2010), and Nurses’ Health Study II (NHS II) (106 122 women, age = 25–42 y; follow-up period from 1991 to 2009) to examine the association between a history of kidney stones and the risk of coronary artery disease. After adjusting for potential confounders, the women with a reported history of kidney stones exhibited an increased risk of CHD compared with those with no history in the NHS I (IR = 754 vs 514 per 100 000 person-y; multivariable HR = 1.18; 95% CI = 1.08–1.28) and NHS II (IR = 144 vs 55 per 100 000 person-y; multivariable HR = 1.48; 95% CI = 1.23–1.78). No significant association was observed for men (IR = 1355 vs 1022 per 100 000 person-y; multivariable HR = 1.06; 95% CI = 0.99–1.13) [39]. The results suggested that compared with men, women had a higher probability of being exposed to unknown factors that could increase their risk of cardiovascular disease and kidney stones [39]. However, their study had some limitations, including the majority of the participants being Caucasian, and the information on stone composition was limited to the cohort.

The pathomechanisms observed in patients with MS regarding the development of endocrine, vasogenic, and renal disarrangement are speculated to be involved in the pathology of ACS and US. After adjusting for medical co-morbidities related to MS, US remains an independent risk factor for subsequent ACS; thus, we hypothesized that traditional herbal medicine consumed by patients with US might contribute to stone formation. Stoller et al hypothesized that the pathogenesis of stones might be vascular injury to the vasa recta near the renal papilla [40]. They asserted that repairing damaged vessel walls could involve an atherosclerotic-like reaction that results in calcification of the endothelial wall, which then erodes into the papillary interstitium and the collecting ducts, contributing to stone formation [40]. The findings of this investigation present a potential relationship between US and ACS, which has not yet been thoroughly examined. Unlike previous studies, we examined not only nephrolithiasis, but also other stone diseases of the ureter and bladder. We believe that these results support the findings of other clinical or basic studies, and recommend further investigation to understand the underling mechanisms.

Although the population-based design is a major strength of this research, several limitations require consideration. First, the diagnoses of US and ACS relied on administrative claims data reported by physicians and hospitals. These might be less accurate than diagnoses made according to standardized criteria. Second, information on the lifestyle or behavior of patients is lacking in the NHIRD; thus, it was impossible to adjust for health- and behavior-related factors such as smoking, alcohol consumption, dietary habits, exercise, and BMI, which are known risk factors for ACS. These behaviors are also indicated to be independent but modifiable risk factors for ACS. However, we lacked information on the chewing of betel quid, which has been reported to increase the incidence of hypercalciuria and hypocitraturia. Third, treatment information is not included in the database, and preventive medication for US-diagnosed patients might interfere with the possible relationship between US and ACS. Finally, this investigation might have been subject to surveillance bias because patients with US typically visit physicians more frequently than other patients do. Thus, their increased exposure to the medical care system could lead to early detection of ACS. This would also contribute to an ascertainment bias, in which patients without US might have been less likely to be included as ACS cases than patients with US were. Despite our meticulous research design and adequate control of confounding factors, a crucial limitation was that biases might have remained because of potential unmeasured or unknown confounders. Overall, our results indicate that patients with US have a comparatively higher risk of developing ACS.

In conclusion, we provide large-scale data evaluating the relationship between US and the risk of developing ACS, particularly among young (≤49 years) and male adults. Future studies should examine age differences as a possible mechanism of US-related ACS morbidity by conducting multicenter recruitment and measurements of laboratory data.
